# Comprehensive analysis of splicing factor SRs-related gene characteristics: predicting osteosarcoma prognosis and immune regulation status

**DOI:** 10.3389/fonc.2024.1456986

**Published:** 2024-09-02

**Authors:** Changhai Long, Biao Ma, Kai Li, Sijing Liu

**Affiliations:** Department of Orthopedic Center, The Second Hospital Affiliated to Guangdong Medical University, Zhanjiang, Guangdong, China

**Keywords:** serine/arginine-rich splicing factor (SRs) related genes, osteosarcoma, SRSF7, T cell, immune microenvironment

## Abstract

**Objective:**

To investigate the impact of SRs-related genes on the overall survival and prognosis of osteosarcoma patients through bulk and single-cell RNA-seq transcriptome analysis.

**Methods:**

In this study, we constructed a prognosis model based on serine/arginine-rich splicing factors (SRs) and predicted the survival of osteosarcoma patients. By analyzing single-cell RNA sequencing data and applying AUCell enrichment analysis, we revealed oncogenic pathways of SRs in osteosarcoma immune cells. Additionally, we described the regulatory role of SRSF7 in pan-cancer.

**Results:**

Lasso regression analysis identified 6 key SRs-related genes, and a prognosis prediction model was established. The upregulation of these pathways revealed that SRs promote tumor cell proliferation and survival by regulating related signaling pathways and help tumor cells evade host immune surveillance. Additionally, by grouping single-cell data using AUCell, we found significant differences in T cell expression between high and low-risk groups. The analysis results indicated that the regulatory activity of SRs is closely related to T cell function, particularly in regulating immune responses and promoting immune evasion. Furthermore, SRSF7 regulates cell proliferation and apoptosis.

**Conclusion:**

SRs-related genes play a critical regulatory role in osteosarcoma. T cells are key in regulating immune responses and promoting immune evasion through SRs genes. SRSF7 is a significant gene influencing the occurrence and development of osteosarcoma.

## 1 Introduction

Osteosarcoma (OS) is the most common primary malignant bone tumor in children and adolescents (1). It is characterized by rapid cell proliferation, high aggressiveness, propensity for metastasis, and pathological bone destruction. In recent years, the application of combined treatment methods, such as preoperative and postoperative chemotherapy and extensive resection, has significantly improved the survival rate (about 60-70%) of patients with osteosarcoma (1–4). However, for patients who develop distant metastases, their five-year survival rate is only 20-28% (5–7). The abnormal regulation of the immune microenvironment within osteosarcoma is closely associated with tumor cell immune evasion, chemotherapy resistance, and metastasis (8, 9). To improve the treatment outcomes of osteosarcoma, it is crucial to identify new therapeutic targets and biomarkers, and to modulate the immune status of osteosarcoma.

Serine/arginine-rich (SR) proteins, members of the RNA-binding protein family, play a key role in the assembly and selective splicing of precursor mRNA (10). Members of the SRSF family control the selective splicing of multiple target genes (11), thereby regulating nearly all critical aspects of tumorigenesis such as cell cycle regulation, apoptosis, genomic stability, cell adhesion and metastasis (12, 13), as well as angiogenesis (14). With the advancement of transcriptome sequencing technology, an increasing number of studies have shown that SR members are overexpressed in cancer tissues. The abnormal alternative splicing events associated with SR overexpression are considered to be one of the important factors leading to the occurrence and development of cancer (15).

The immune microenvironment refers to a complex network surrounding the tumor, which includes immune cells, inflammatory mediators, and other immune-related molecules (16–18). The interaction between the immune system and tumor cells undergoes different stages, including immune elimination, equilibrium, and escape (19–21). In osteosarcoma, tumor cells release new antigens or tumor-associated antigens, which are captured and presented to activate cytotoxic T cells (CTLs), thereby initiating immune attack to clear tumor cells. Activated CTLs enter the tumor microenvironment, eliminate tumor cells, and lead to the release of more antigens, further activating the immune response. Several immune escape mechanisms have been discovered so far, including antigen loss, tumor-induced immune suppression, tumor cell evasion, lack of co-stimulatory signals on the surface of tumor cells, and tumor cell resistance to apoptosis. Specifically, tumors establish complex negative feedback loops by releasing immune inhibitory factors and activating immune checkpoint molecules (such as PD-L1, CTLA-4, etc.), thereby effectively evading T cell-mediated immune attacks (22). An increasing number of studies have revealed that RNA splicing and modifications affect the formation of the tumor immune microenvironment and the immune evasion ability of tumor cells. For example, METTL3 promotes the formation of circ-IGF2BP3 in a YTHDC1-dependent manner, protecting PD-L1 from proteasome-mediated degradation, thereby reducing CD8+ T cell infiltration and promoting immune evasion of non-small cell lung cancer (NSCLC) cells. Additionally, N6-methyladenosine (m6A) modification is positively correlated with the number of CD8+ T cells in the immune microenvironment of pancreatic cancer, suggesting that m6A modification may play an important role in promoting the aggregation and activation of CD8+ T cells. Therefore, the regulation of key molecules in RNA splicing and modification processes plays an important role in the immune microenvironment of osteosarcoma.

In this study, osteosarcoma patients were divided into two subtypes based on the expression of Serine/Arginine-Rich Splicing Factor (SR) related genes. The differences in patient prognosis and tumor immune microenvironment between these two subtypes were investigated using Lasso machine learning. Additionally, this study subgrouped osteosarcoma single-cell samples based on SR-related genes to explore the regulatory patterns of transcription factors and immune-infiltrating cells. Finally, the genes of interest in SRs were validated through *in vitro* functional experiments and pan-cancer analysis.

## 2 Materials and methods

### 2.1 Data download

We downloaded TARGET data from the UCSC XENA database, acquiring RNA-seq data and associated clinical characteristics for 84 osteosarcoma tissue samples. We obtained data for 12 SR splicing factor-related genes (SRSF1, SRSF2, SRSF3, SRSF4, SRSF5, SRSF6, SRSF7, SRSF8, SRSF9, SRSF10, SRSF11, and SRSF12). Additionally, single-cell RNA sequencing (scRNA-seq) data from 6 osteosarcoma patients in the GEO dataset were utilized (GSE162454). Expression and clinical data from TCGA Pan-Cancer and GTEx were downloaded from the UCSC XENA database (https://xenabrowser.net/datapages/).

### 2.2 Construction of the prognostic model for the SRSF family

The iterative Least Absolute Shrinkage and Selection Operator (LASSO) Cox regression model was used to identify the optimal gene features of the SR family in osteosarcoma (OS). An SR-related score was then constructed using the coefficients of the identified gene features. The median SR-related score was used as a cutoff to divide patients into high SR score and low SRSF score groups. The SR-related score was calculated using the following formula: SRSF score = Σ (Coef_i × Exp_i), where Coef_i represents the coefficient and Exp_i represents the expression level of each gene in the signature. Kaplan-Meier analysis was performed to compare survival differences between the high and low SRSF score groups. A stratified analysis was conducted to assess whether SRs-related scores are independent prognostic factors for OS. The performance of the classifier was evaluated using the area under the curve (AUC) from the “timeROC” package in R. Additionally, the prognostic value of the genes in the model was assessed.

### 2.3 Functional and pathway enrichment analysis

To identify differentially expressed genes (DEGs), we analyzed RNA-seq data from both high-risk and low-risk osteosarcoma patient groups. DEGs were determined using the DESeq2 package in R. The criteria for DEGs were set with a false discovery rate (FDR) < 0.05 and a log2 fold change (log2FC) > 1 or < -1. The total number of samples used for this analysis included 84 osteosarcoma tissue samples from the TARGET database. To explore the functional enrichment differences between high and low-risk groups, we conducted Kyoto Encyclopedia of Genes and Genomes (KEGG) analysis on the identified DEGs. This analysis was performed using the Gene Set Enrichment Analysis (GSEA) function of the clusterProfiler R package. The network diagram was constructed using the aPEAR R package. We downloaded the Hallmark gene set, C2.CP.KEGG_Legacy.v2023.2.Hs.symbols, and C5.all.v2023.2.Hs.symbols from the Molecular Signatures Database (MSigDB). Immune pathway analysis was conducted using datasets collected with the IOBR R package. Correlation heatmaps and dot plots were generated using the ggplot2 package.

### 2.4 Single-cell RNA sequencing data processing and analysis

The single-cell RNA sequencing (scRNA-Seq) data from GEO database accession GSE162454 includes samples from 6 primary osteosarcoma tumors. Subsequently, we performed preprocessing of the normalized scRNA-Seq data using the R software package “Seurat”. To obtain high-quality single-cell data, we filtered out genes expressed in fewer than three cells, cells with detected gene counts fewer than 500 or more than 6000, and cells with mitochondrial content exceeding 10%. Before removing batch effects and performing dimensionality reduction using Principal Component Analysis (PCA) and Uniform Manifold Approximation and Projection (UMAP), single-cell RNA sequencing data were standardized using LogNormalize. The cell clustering was performed using the “FindClusters” function from the R package “Seurat”. Cell annotations were derived from previous studies. For visual representation of the results, we utilized t-distributed Stochastic Neighbor Embedding (t-SNE) to reduce the complexity of the data. The RunTSNE function was employed to generate a two-dimensional t-SNE plot based on the top 30 principal components. Subsequently, the t-SNE plot was generated using the scCustomize R package. The “FindAllMarkers” function was utilized to identify differentially expressed genes (DEGs) between subgroups, with thresholds set at log2 fold change (|FC|) > 0.5 and *P* value < 0.05. The differential gene expression data from time-series analysis were clustered and visualized using the fuzzy c-means algorithm in the R package ClusterGVis. Additionally, we presented the top 5 marker genes and utilized the enrichCluster R package to demonstrate the biological process pathways for each cell cluster.

### 2.5 SRs modify activity scores

We evaluated the SRs modification activity scores for six significantly identified candidate splicing factor genes (SRSF1, SRSF4, SRSF5, SRSF7, SRSF8, and SRSF10) using the AUCell R package. We computed the area AUC values using AUCell for the set of six genes. This metric ranks gene expression within each cell, reflecting the proportion of highly expressed genes in the gene set for that cell. To distinguish active gene sets, we utilized the “AUCell_exploreThresholds” function to determine a threshold (0.04). Finally, we employed t-distributed stochastic neighbor embedding (t-SNE) to visualize the AUC scores of each cell, depicting which cells are in an active state.

### 2.6 Subtype analysis and feature enrichment

To comprehensively assess the regulatory network activity of transcription factors (TFs) in single-cell RNA sequencing (scRNA-seq) data, we utilized the PySCENIC framework for integrated enhancer analysis (23). Specifically, we employed the PySCENIC Conda environment and utilized log-transformed expression counts of osteosarcoma cells as input data for initial regulatory network construction. The identification of transcription factors (TFs) was based on the human TF list compiled by Lambert et al. and was performed using the default parameters of PySCENIC. To refine the TF-target gene interactions within the regulons, we integrated the CisTARGET database (24). This database identifies potential TF binding sites by analyzing DNA sequences within 500 base pairs of transcription start sites, as well as within 5Kb and 10Kb intervals. The purpose of this step is to utilize known human TF motif information to further elucidate the regulatory relationships between TFs and their target genes. During this process, we also employed a Drop-out masking strategy to mitigate the impact of common data loss in single-cell data on the analysis results. Finally, utilizing the FindAllMarkers function provided by R language, we identified differentially expressed genes across various groups and cell types. Subsequently, we performed enrichment analysis on these genes to elucidate the functional significance of TF regulatory networks in different cellular states and biological processes.

### 2.7 Reference mapping

The reference atlas of tumor-infiltrating T lymphocytes was loaded from the ProjecTILs Git repository. Additionally, the ProjecTILs R package (version 3.0.0) was used to map the scRNA-seq data onto the reference CD4+ T cell and CD8+ T cell atlases (25, 26). This reference-based analytical approach facilitated the classification and comparison of T cell state distributions. Furthermore, stacked bar plots were employed to illustrate the changes in different cell type states.

### 2.8 Trajectory analysis in Monocle3

To understand the potential pseudotemporal relationships between different cell types, we performed trajectory analysis using Monocle3 (27). The Seurat object was split into Active and Inactive states from the integrated data, followed by subset analysis of all cell types. The Seurat object was converted to a Monocle3 object using the ‘as.cell_data_set’ function from SeuratWrappers. Subsequently, pseudotime analysis was conducted on the Monocle3 object utilizing the ‘learn_graph’ and ‘order_cells’ functions.

### 2.9 Immune infiltration analysis

Immune infiltration analysis was performed using the “IOBR” package (28), employing the EPIC algorithm to compare differences between high-risk and low-risk groups. Additionally, immune infiltration was assessed using the CIBERSORT and Estimate algorithms, followed by correlation analysis.

### 2.10 Cell culture

The MG63 and SJSA-1 cell lines were obtained from Procell (Wuhan, China). These cells were cultured in DMEM medium supplemented with 10% fetal bovine serum and antibiotics (100 U/mL penicillin and 10 mg/L streptomycin). The cells were maintained in an incubator at 37°C with 5% CO_2_.

### 2.11 SiRNA transfection

SRSF7 siRNA and the corresponding si-control were purchased from Guangzhou RiboBio Co., Ltd. MG63 and SJSA-1 were transfected using Lipo8000 (Beyotime, Shanghai) following the manufacturer’s instructions. After 24 hours post-transfection, cells were used for protein quantification. The sequence of SRSF7 siRNA is as follows: Sense (sense strand): 5’-GUGCAAGUCCUGAAAGAAU-3’, Antisense (antisense strand): 5’-AUUCUUUCAGGACUUGCACT-3’.

### 2.12 Cell viability assay

Transfected cells were cultured in 96-well plates at a density of 5000 cells per well. Cells were treated with Cell Counting Kit-8 (CCK-8) reagent (Beyotime, Shanghai) and incubated at 37°C before detection. The absorbance at 450 nm was measured using a microplate reader at 24, 36, and 72 hours. To assess the colony-forming ability of osteosarcoma cells, a plate colony formation assay was performed. Transfected cells were evenly seeded in six-well plates, cultured for 12 days with regular medium changes. Fixed and stained using paraformaldehyde and crystal violet staining solution. Cell images were captured using a digital camera and data were recorded.

### 2.13 Detection of cell apoptosis

The Annexin V-FITC apoptosis detection kit was purchased from Beijing Solaibao Technology Co., Ltd. Human osteosarcoma cells (MG63 and SJSA-1 cell lines) in logarithmic growth phase were seeded into 6 cm culture plates. After 24 hours of culture, cells were treated with si-SRSF7 for 24 hours. Cells were digested with trypsin without EDTA and collected. After washing twice with 4°C PBS, cells were resuspended in 1x binding buffer and then suspended at a density of 10^6 cells/mL in 100 μL of binding buffer. Subsequently, 5 μL of Annexin V/FITC (Beyotime, Shanghai) was added and gently mixed. Cells were incubated at 4°C in the dark for 5 minutes. After incubation, 5 μL of PI was added, gently mixed, followed by the addition of 400 μL of PBS. Cell apoptosis analysis was performed using a flow cytometer.

### 2.14 Western blotting

In brief, proteins were first separated using SDS-PAGE (Epizyme, Shanghai). Subsequently, proteins from the gel were transferred onto a PVDF membrane and blocked. Primary antibodies were incubated overnight at 4°C. The following day, the membrane was incubated with secondary antibodies. After washing three times with TBST, the membrane was incubated with enhanced chemiluminescence (ECL) substrate for detection (Biosharp, Beijing).

### 2.15 Statistical analysis

We used the Wilcoxon rank sum test or the KruskalWallis test to determine differences between groups. A two-sided *P* value of < 0.05 was considered statistically significant.

## 3 Results

### 3.1 Construction and validation of a SRs prognostic signature for OS

**Figure 1:**
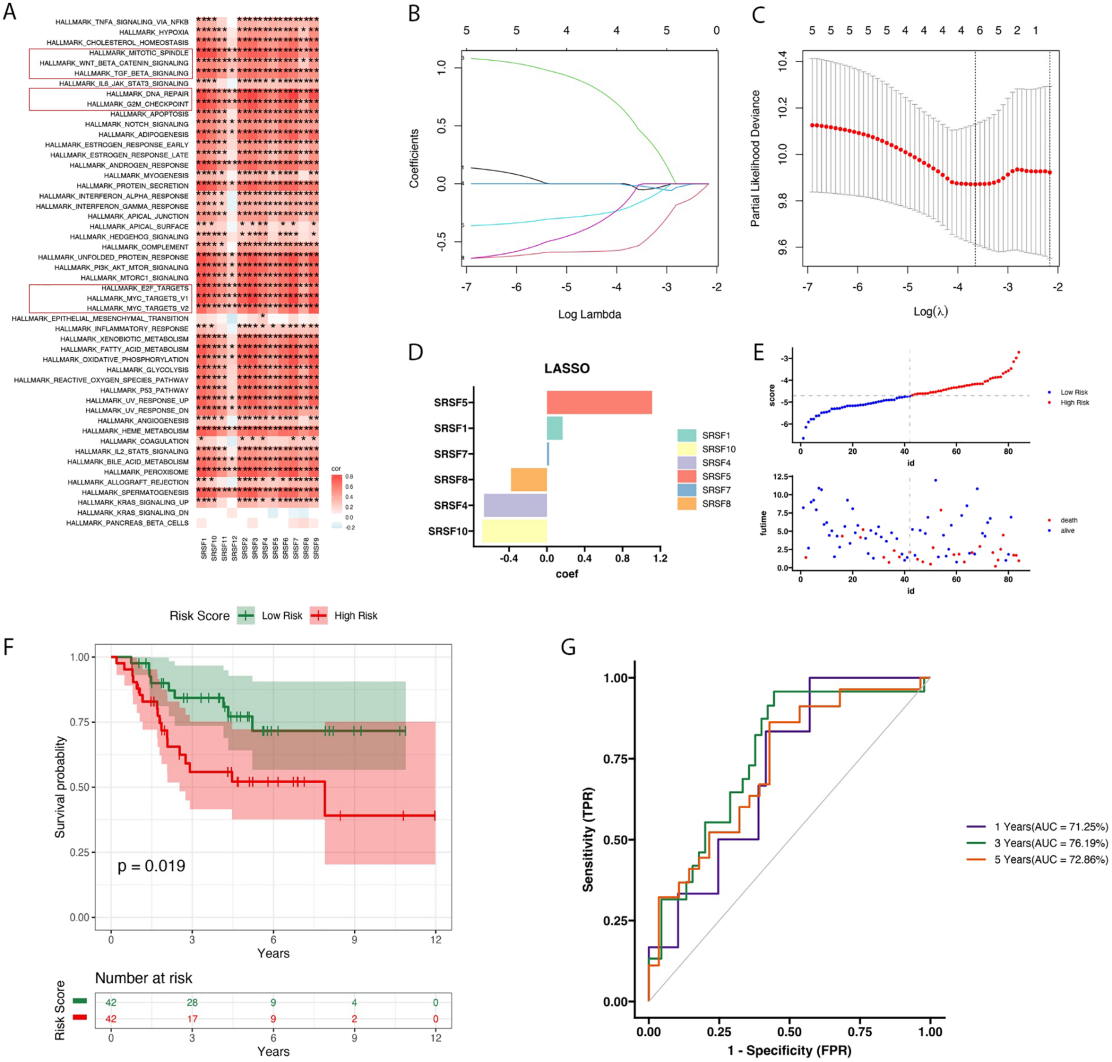
Heatmap of SRs expression in Hallmark pathways, LASSO regression model analysis, and prognosis feature evaluation. **(A)** The heatmap displayed the enriched expression of SRs (relevant receptors) in Hallmark pathways along with the correlation analysis. Pearson correlation analysis was used to evaluate the correlation between pathway and gene expression. The heatmap colors transition from blue (indicating low correlation, r = -1) to red (indicating high correlation, r = 1). **(B)** The LASSO regression model was used to determine the optimal regularization parameter γ. **(C)** Variations in LASSO coefficients of SRs under different regularization parameters. **(D)** The bar graph depicting the coefficients (Coef) values of six features significantly associated with prognosis. **(E)** The scatter plot divided patients into high and low-risk subgroups based on the median, highlighting that death events predominantly occurred in the high-risk group. **(F)** he survival analysis plot revealed that patients in the high-risk group exhibited poorer prognosis compared to those in the low-risk group, as reflected in overall survival rates. **(G)** Using the TARGET dataset, time-dependent ROC curves were plotted to demonstrate the accuracy of survival prediction at 1 year, 3 years, and 5 years, with survival rates of 71.25%, 76.19%, and 72.86%, respectively.

In osteosarcoma data, we found that 12 splicing factor genes were associated with multiple pathways in the Hallmark gene set, including the EF2, G2M, WNT, DNA repair, and MYC pathways (**Figure 1A**). Subsequently, we further screened out the most significant candidate genes using LASSO regression and constructed a prognostic model to predict the prognosis of osteosarcoma patients (**Figures 1B, C**). We identified 6 splicing factor genes associated with the prognosis of osteosarcoma patients, namely SRSF1, SRSF4, SRSF5, SRSF7, SRSF8, and SRSF10. Next, we divided the training cohort into two groups based on the risk score. The risk score was calculated as follows:

RiskScore=0.165×SRSF1−0.665×MYC+1.111×SRSF5 + 0.022×SRSF7−0.381×SRSF8−0.685×SRSF10. Additionally, we analyzed the distribution of risk scores and survival status based on the candidate gene expression pattern (**Figures 1D, E, G**). The Kaplan-Meier curve showed that with an increase in the risk score, the survival time of patients in the training set significantly decreased. Additionally, patients in the high-risk group had significantly worse prognosis compared to those in the low-risk group (*P*=0.019, **Figure 1F**). Furthermore, we conducted ROC curve analysis to evaluate the performance of the prognostic model. The AUC for the first year, third year, and fifth year were 71%, 76%, and 72%, respectively. These results indicate that the prognostic model we constructed has a certain accuracy and reliability in predicting the survival of osteosarcoma patients.

### 3.2 Enrichment analysis and immune infiltration in high and low-risk groups

**Figure 2:**
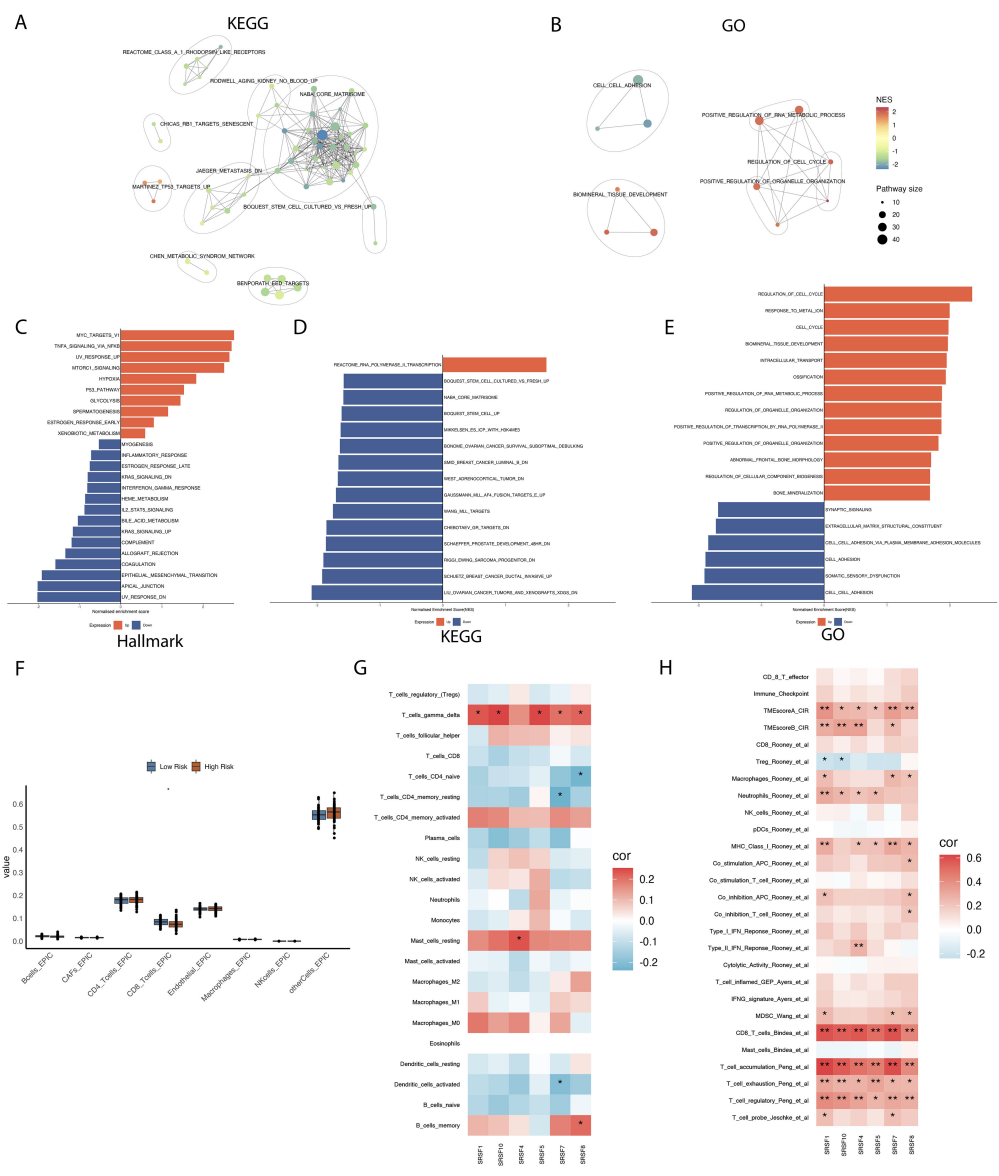
Enrichment analysis of differential genes in high- and low-risk groups, and their correlation with immune cell infiltration and prognosis characteristics. **(A)** The enrichment network diagram reveals clustering analysis results of differential genes between high and low risk groups in the Kyoto Encyclopedia of Genes and Genomes (KEGG) dataset. **(B)** The enrichment network diagram provides detailed clustering analysis results of differential genes between high and low risk groups in the Gene Ontology (GO) dataset. **(C-E)** The bar charts were used to compare and illustrate enrichment analysis results of differential genes between high and low risk groups across Hallmark, Gene Ontology (GO), and Kyoto Encyclopedia of Genes and Genomes (KEGG) datasets. **(F)** The box plots illustrate the distribution differences in immune cell infiltration between high- and low-risk groups. **(G, H)** The correlation between six prognostically significant features (SRs) and immune cells, as well as their associated immune pathways. *p < 0.05, ** p < 0.01.

To further understand the roles of high and low-risk groups in osteosarcoma, we conducted enrichment analyses of differentially expressed genes between these groups using various gene sets. In the enrichment analysis of the Hallmark gene set (**Supplementary Table 1**), we observed upregulation in pathways such as MYC_TARGETS_V1, TNFA_SIGNALING_VIA_NFKB, HYPOXIA, and MTORC1_SIGNALING in the high-risk group (29) (**Figure 2C**). These findings indicate an active state of tumor cells in biological processes such as proliferation, metabolism, angiogenesis, and metastasis (30–32). Additionally, we observed downregulation of immune response-related pathways in the high-risk group, such as INFLAMMATORY_RESPONSE, INTERFERON_GAMMA_RESPONSE, IL2_STAT5_SIGNALING, COMPLEMENT, and ALLOGRAFT_REJECTION. These results suggest impaired immune system function and reduced immune responses in the high-risk group, enabling tumor cells to evade immune surveillance more easily. In the KEGG results (**Figures 2A, D**; **Supplementary Table 2**), we observed upregulation of the REACTOME_RNA_POLYMERASE_I_TRANSCRIPTION pathway in the high-risk group. Excessive activity of RNA polymerase I may lead to increased synthesis of rRNA, thereby promoting the growth and proliferation of tumor cells (33, 34). Additionally, we observed downregulation of the NABA_CORE_MATRISOME pathway in the high-risk group. This pathway includes genes encoding core extracellular matrix components such as ECM glycoproteins, collagens, and proteoglycans (35, 36). Downregulation of this pathway may affect the structural and functional integrity of the extracellular matrix, potentially influencing the adhesion, migration, and invasion capabilities of tumor cells. GO enrichment results (**Figures 2B, E**; **Supplementary Table 3**) indicate upregulation of pathways such as POSITIVE_REGULATION_OF_RNA_METABOLIC_PROCESS, REGULATION_OF_ORGANELLE_ORGANIZATION, and POSITIVE_REGULATION_OF_TRANSCRIPTION_BY_RNA_POLYMERASE_II in the high-risk group. These may reflect the tumor cells’ enhanced control over gene expression and organelle function, providing the necessary materials and energy for tumor growth and survival.

### 3.3 SRs are associated with cancer immunity

In recent years, the intricate relationship between cancer and immunity has been increasingly recognized. Given the widespread genomic alterations and expression disorders of SRSFs in various types of cancer, and their close association with multiple carcinogenic pathways, particularly those related to immunity, we are interested in further exploring the potential connections between SRSFs and cancer immunity. We conducted an Epic immune infiltration analysis (**Figure 2F**), which revealed significant differences in CD8^+^ T cells between the high-risk and low-risk groups (*P* value=0.0302). Additionally, using the CIBERSORT algorithm to evaluate various immune cell types, we found significant differences in T cell gamma delta levels between groups. However, CD8+ T cells did not show significant variation in this broader immune profiling. This indicates that T cell gamma delta may play a distinct or additional role in the osteosarcoma immune microenvironment (**Figure 2G**). Further analysis within T cell immune pathways (**Figure 2H**) demonstrated a high correlation of these splice factor genes with the CD8^+^ T cell immune pathway. These findings suggest a linkage between high and low risk groups in terms of immune infiltration, splice factor gene expression, and T cell immune pathways, which may influence the progression of osteosarcoma and patient prognosis.

### 3.4 Single-cell data dimensionality reduction clustering annotation results

**Figure 3:**
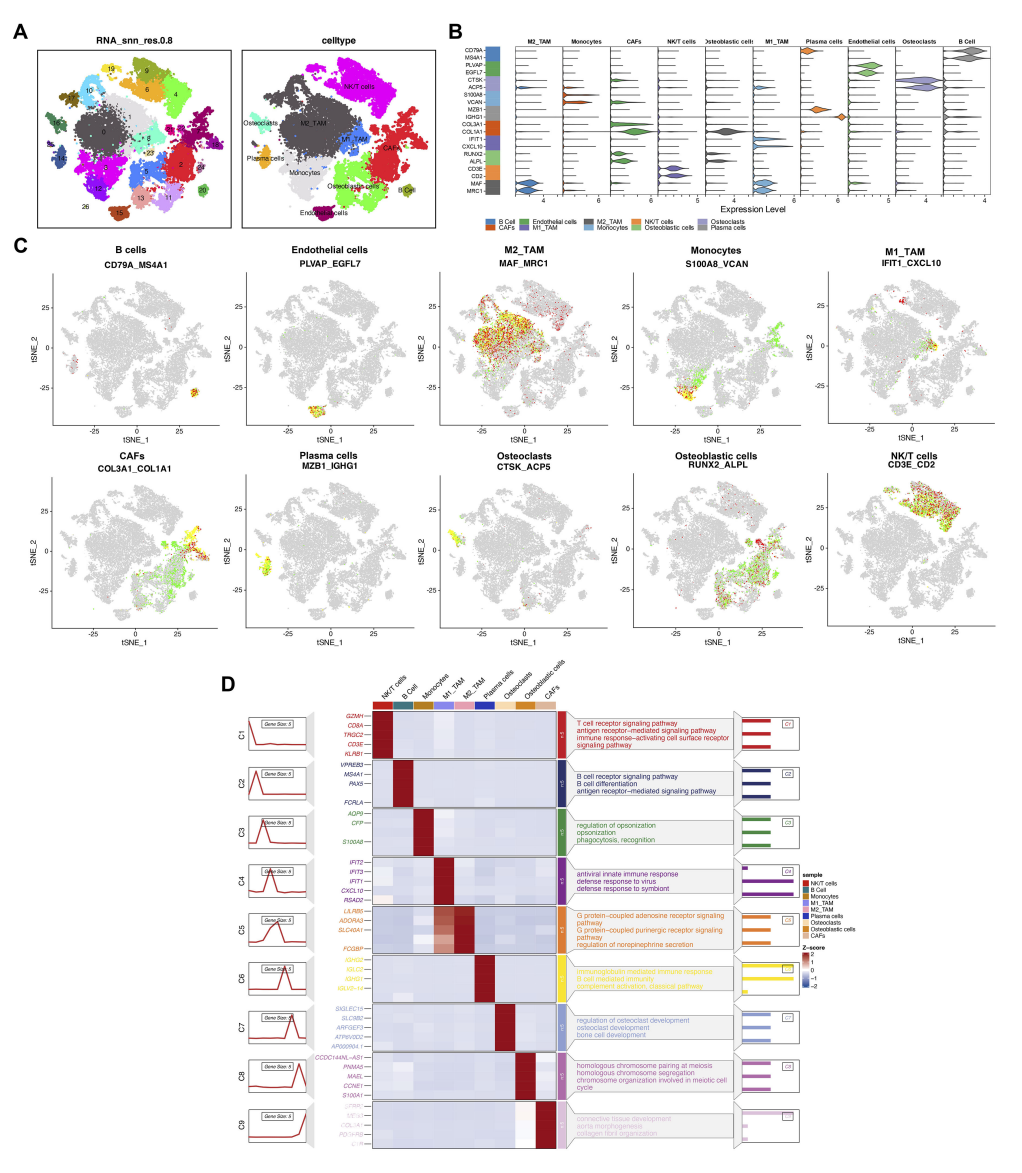
Multidimensional analysis of cellular heterogeneity and functional characteristics. **(A)** The t-distributed stochastic neighbor embedding (t-SNE) plot illustrates clustering results based on transcriptomic data and annotates cellular populations, revealing cellular heterogeneity within the samples. Different colors represent distinct cellular populations, displaying the distribution and interrelationships of cell types. **(B)** The violin plot provides detailed expression profiles of cell type-specific marker genes. **(C)** The t-SNE plot shows spatial expression distributions of key markers (CD79A, MS4A1, PLVAP, EGFL7, MAF, MRC1, S100A8, VCAN, IFIT1, CXCL10, COL3A1, COL1A1, MZB1, IGHG1, CTSK, ACP5, RUNX2, ALPL, CD3E, CD2) across all cells. **(D)** The heatmap reveals gene expression patterns associated with various biological processes and pathways across different cellular populations. The color gradient represents the z-score of gene expression, with the sidebar indicating enrichment pathways for specific cellular populations.

We utilized t-SNE to visualize clustering results, grouping all cells from six osteosarcoma samples in the database into 26 distinct cell clusters (**Figure 3A**). To clarify the type of each cluster, we annotated each cell subgroup using cell marker genes from published literature. The results indicated that these 26 cell subclusters were annotated as ten different cell types: NK/T cells, M2-type tumor-associated macrophages, osteoclasts, cancer-associated fibroblasts, M1-type tumor-associated macrophages, monocytes, osteoblasts, B cells, endothelial cells, and plasma cells. The violin plot displays the expression of marker genes across different cell types, with each marker gene showing elevated expression levels in its corresponding cell type (**Figure 3B**). Specifically, CD79A and MS4A1 are highly expressed in B cells; PLVAP and EGFL7 in endothelial cells; MAF and MRC1 in M2-type tumor-associated macrophages; S100A8 and VCAN in monocytes; IFIT1 and CXCL10 in M1-type tumor-associated macrophages; COL3A1 and COL1A1 in cancer-associated fibroblasts; MZB1 and IGHG1 in plasma cells; CTSK and ACP5 in osteoclasts; RUNX2 and ALPL in osteoblasts; CD3E and CD2 in NK/T cells (**Figure 3C**). Based on the enrichment analysis of highly expressed genes in each cell cluster, we further validated the reliability of the cell cluster annotations. The results showed that NK/T cells were enriched in the T cell receptor signaling pathway; B cells were enriched in the B cell receptor signaling pathway; osteoclasts were enriched in osteoclast development (**Figure 3D**).

### 3.5 Single-cell data identifies active and inactive SRSFs shear factor cells in osteosarcoma

**Figure 4:**
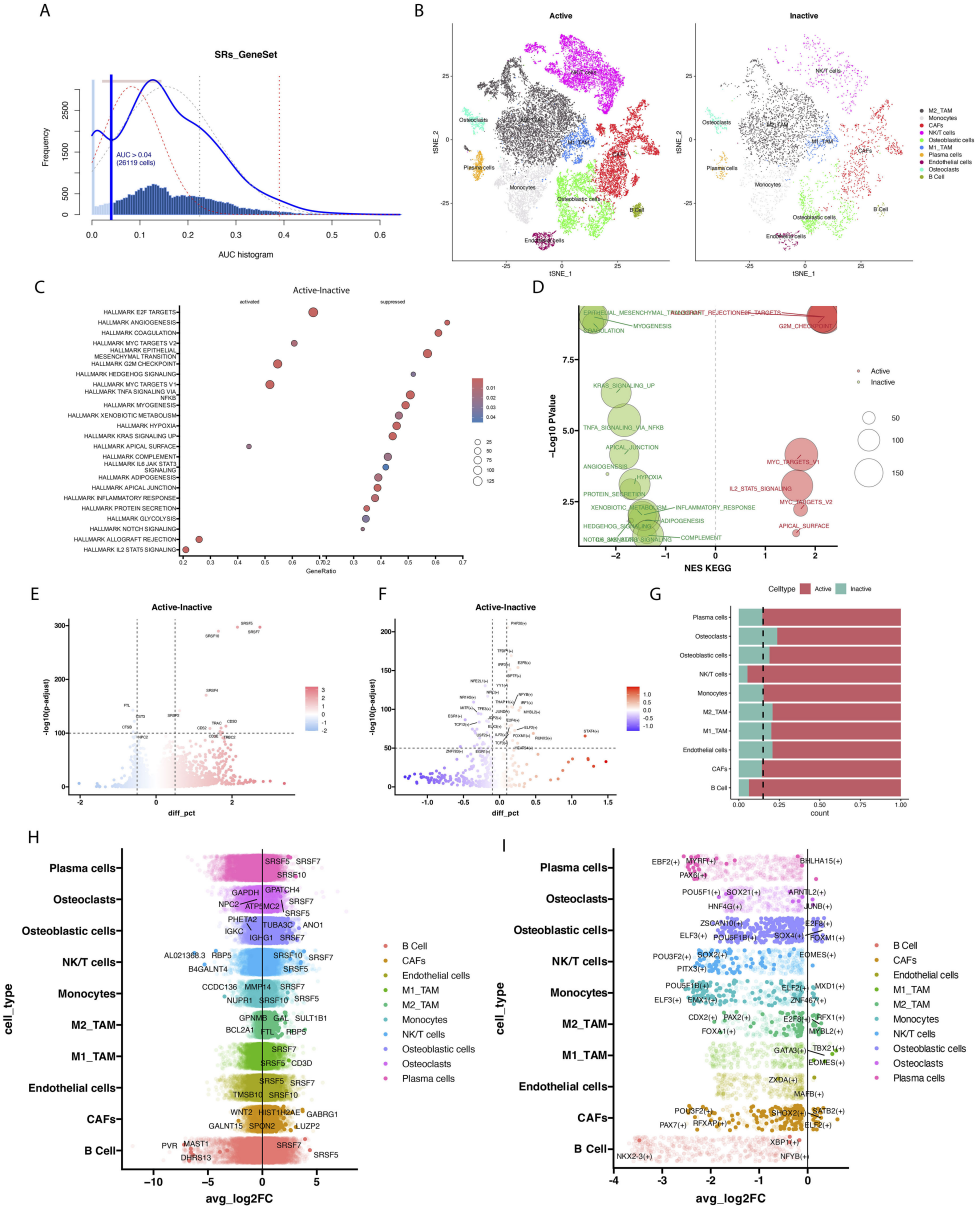
The modification activity of SRs influences cellular epigenetic characteristics and functions. **(A)** The scores of modification activity for 6 SRs. The threshold is chosen such that SRs modification scores above the threshold (represented by dashed lines fitting Gaussian distributions for each distribution). **(B)** The t-SNE plot illustrates cellular grouping based on SRs modification activity, categorizing cells into Active and Inactive groups. **(C)** The scatter plot displays enrichment of Hallmark pathways between cells with active SRs modification and those with inactive SRs modification. **(D)** The scatter plot describes enrichment of KEGG pathways between cells with active SRs modification and those with inactive SRs modification. **(E)** The volcano plot displays significant differences in gene expression between cells with active SRs modification and cells with inactive SRs modification. In the volcano plot, each point represents a gene, with the x-axis indicating the magnitude of gene expression difference and the y- axis representing statistical significance. **(F)** The volcano plot illustrates significant differences in transcription factor expression between cells with active SRs modification and those with inactive SRs modification. **(G)** Stacked bar chart showing the proportion changes of different cell populations between Active and Inactive states. **(H)** The volcano plot illustrates significant differences in transcription factor expression between cells with active SRs modification and those with inactive SRs modification. **(I)** The bar volcano plot displays genes with high and low expression across various cellular populations. Each bar represents a gene, with its height indicating its expression level within that cellular population.

We used AUCell to score the SRSFs set within individual cells to further understand SRSFs activity. All cells showed two peaks in AUCell values, with 26,119 cells displaying relatively high AUC values when the AUC threshold was set to 0.04 (**Figure 4A**). Subsequently, we divided the immune cells of osteosarcoma into two different groups based on splicing factor modification (**Figure 4B**): SRs active (AUC value > 0.04) and SRSFs inactive (AUC value < 0.04). We performed differential gene analysis on cells with different SRSFs activities and observed 10 upregulated genes and 4 downregulated genes (|logFC| > 0.5 and *P* value < 1e-100, **Figure 4E**).

In our study, the upregulated genes observed mainly included members of the splicing factor family such as SRSF7, SRSF5, SRSF10, SRSF4, and SRSF2, as well as key marker genes of T cells such as CD52, TRAC, CD3D, CD3E, and TRBC2. The enhanced expression of these upregulated genes may reflect the activation of splicing regulatory mechanisms and the enhancement of T cell activity. In contrast, the downregulated genes included FTL, CST3, CTSB, and NPC2. The decreased expression of these genes may indicate abnormalities in iron metabolism, weakened lysosomal function, or inadequate intracellular cholesterol transport. Moreover, analysis of different cell clusters showed frequent occurrences of SRSF7, SRSF5, and SRSF10 in multiple clusters (**Figure 4H**). To fully understand the complexity of SRSFs (splicing regulatory proteins) modifications, it is necessary to complement gene expression analysis by understanding potential gene regulatory networks. In this regard, we performed transcription factor differential analysis between cell types with different SRSFs activities and non-activities using the transcription factor-based gene regulatory network PySCENIC (**Figures 4F, I**). For instance, the transcription factor SOX2 may play a regulatory role in tumor immunity, particularly in association with T cell immunity. Antibodies against SOX2 have been detected in some small cell lung cancer patients, which may be associated with better prognosis. STAT4 is considered a key molecule that drives optimal antigen-specific responses and can overcome STAT1-dependent inhibition, thereby promoting cell proliferation. Furthermore, pathway enrichment analysis of differentially expressed genes was performed to reveal the potential roles of these genes in cellular biological processes. The analysis results (**Figure 4C**) showed enhanced activity in the E2F TARGETS, MYC TARGETS, and G2M CHECKPOINT pathways, suggesting that these pathways may play a key role in promoting cell cycle progression and increasing cell proliferation and division activities. In addition, pathways such as ANGIOGENESIS, COAGULATION, Epithelial-Mesenchymal Transition (EMT), TNF-α signaling (TNFA), and response to hypoxia exhibited a downregulation trend. Although the downregulation of these pathways theoretically may inhibit tumor growth, the robust cell cycle-promoting signals under the activation status of splicing factors may have overridden these inhibitory effects. This indicates that even in the suppression of biological processes such as angiogenesis and inflammatory response, signals promoting cell cycle and proliferation may still be the major driving force behind tumor growth. Through KEGG enrichment analysis (**Figure 4D**), our study revealed that the T_CELL_RECEPTOR_SIGNALING_PATHWAY, SPLICEOSOME, and CELL_CYCLE were upregulated, while LYSOME and COMPLEMENT_AND_COAGULATION_CASCADES were downregulated. This further confirms the crucial role of the SR protein family in immune cells by regulating cell proliferation and alternative splicing processes. Particularly, the significant enrichment of the T cell receptor pathway not only emphasizes the central role of T cells in the SRSFs regulatory network but also underscores the importance of SRs in cellular immune responses.

### 3.6 NK/T cell re-annotation

**Figure 5:**
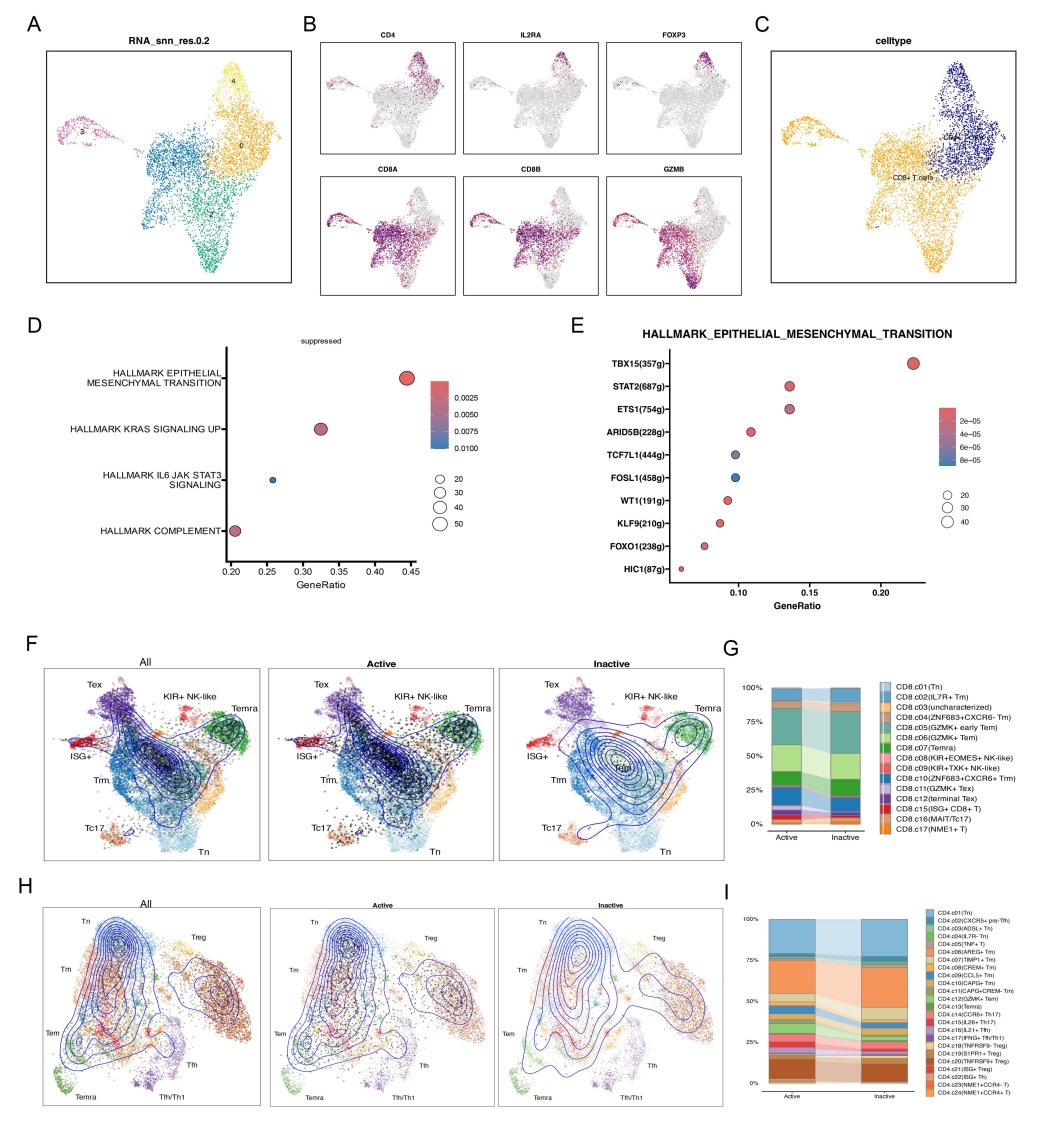
Re-distribution and mechanisms of CD4+ and CD8+ T cells. **(A)** t-SNE plot showing the clustering results based on NK/T cell transcriptome data. **(B)** t-SNE plot showing the spatial expression distribution of key markers (CD4, IL2RA, FOXP3) in all cells. **(C)** t-SNE plot showing CD8+ T cell and CD4^+^ T cell clusters. **(D)** Scatter plot showing the enrichment of Hallmark pathways in Active and Inactive CD8^+^ T cells. **(E)** Scatter plot showing the GRN transcription factor enrichment analysis for the EMT pathway. **(F-I)** ProjecTIL analysis of CD8^+^ T cells and CD4^+^ T cells, illustrating the projection of Active and Inactive cell types onto the reference atlas and the stacked bar plots depicting the cell subtype distribution within each CD8^+^ T cell and CD4^+^ T cell subtype.

In both the Active and Inactive groups, we found that NK/T cells and B cells were the most activated; thus, we conducted further analysis on NK/T cells (**Figure 4G**). Using TSNE dimensionality reduction analysis, NK/T cells were re-clustered into 5 cell clusters (**Figure 5A**). Based on this analysis, we further annotated NK/T cells accurately, delineating specific cell subtypes. Specifically, CD4, IL2RA, and FOXP3 were identified as marker genes for CD4^+^ T cells, while CD8A, CD8B, and GZMB served as markers for CD8^+^ T cells (**Figure 5B**). Through this approach, NK/T cells were ultimately classified into two major categories: CD4^+^ T cells and CD8^+^ T cells (**Figure 5C**). Cell state annotation was automatically performed using the ProjecTILs method with a reference projection. Upon further annotation analysis of CD8+ T cells, we found that cells in an inactive state were mainly concentrated in subtypes such as GZMK^+^ early Tem, GZMK^+^ Tem, Temra, ZNF683^+^ CXCR6^+^ Trm, and others. In contrast, CD8^+^ T cells in an active state tended to be classified as GZMK^+^ Tex, terminal TEX, ISG^+^CD8^+^ T cells, and similar types (**Figures 5F, G**). Similarly, in the annotation of CD4^+^ T cells, cells in an inactive state were mainly distributed in subtypes such as Tn, AREG^+^ Tm, TNFRSF9^+^ Treg, and others. In the Active group, we observed a relatively decreased proportion of CD4^+^ T cell types compared to the Inactive group (**Figures 5H, I**). Meanwhile, there was an increased proportion of subtypes such as ISG^+^ Treg and GZMK^+^ Tem. In CD8^+^ T cells, the Active group was enriched in pathways associated with inflammation suppression. Through transcription factor regulation prediction, we found that transcription factors such as TBX15 (+), STAT2 (+), and FOSL1 (+) were involved in regulating this pathway (**Figures 5D, E**).

### 3.7 Monocle3 analyzes active and inactive

**Figure 6:**
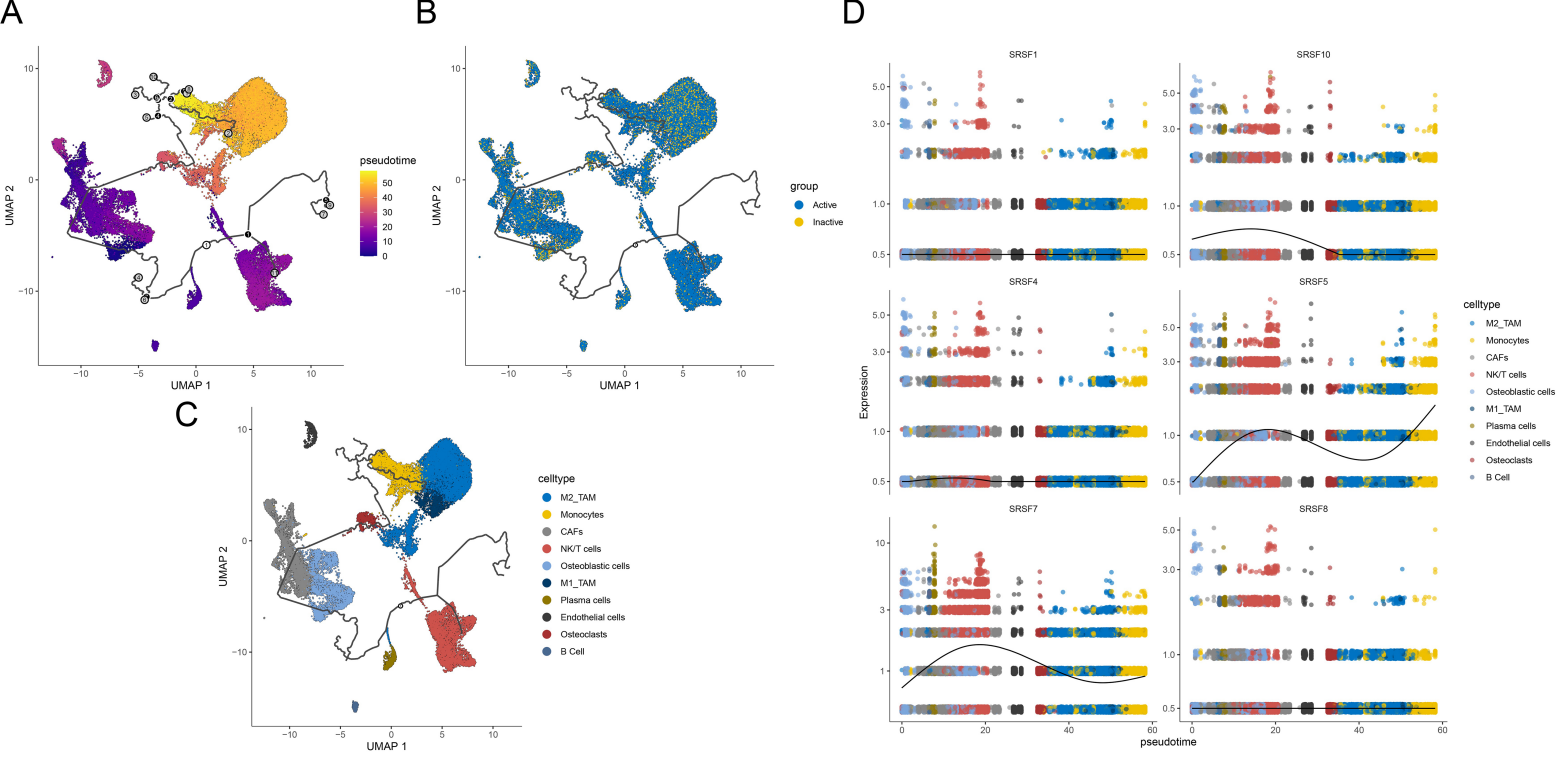
Trajectory reconstruction reveals differential activation between Active and Inactive states in osteosarcoma. **(A)** UMAP plot showing trajectory reconstruction with colors ranging from purple to yellow representing different pseudotime cell states, reflecting temporal progression. **(B)** UMAP plot indicating cell states with yellow denoting Inactive and blue denoting Active states. **(C)** UMAP plot distinguishing various cell types with different colors. **(D)** Pseudotime representation of SRs between Active and Inactive cell states. The x-axis represents cell state progression over the trajectory, while the y-axis displays gene expression values, highlighting the differences in SR and prognostic gene activation between Active and Inactive states.

In our study, we performed pseudotime analysis starting from the Inactive group to reveal the dynamic changes of different cell types during differentiation. We found that NK/T cells, osteoblasts, and CAFs were in the early stages of differentiation, while over time, osteoclasts, M1_TAMs, M2_TAMs, and monocytes gradually transitioned to late-stage differentiation (**Figures 6A-C**). Particularly, the study of the expression and evolutionary status of six SRs splicing factors (SRSF1, SRSF4, SRSF7, SRSF8, SRSF5, SRSF10) during this differentiation process revealed that the expression levels of these splicing factors were higher in the NK/T cell stage, mainly concentrated in the early stages of differentiation. As differentiation progressed, the expression levels of these splicing factors gradually decreased, with a significant decrease observed in SRSF7, SRSF5, and SRSF10 (**Figure 6D**).

### 3.8 Pan-cancer analysis of SRSF7

**Figure 7:**
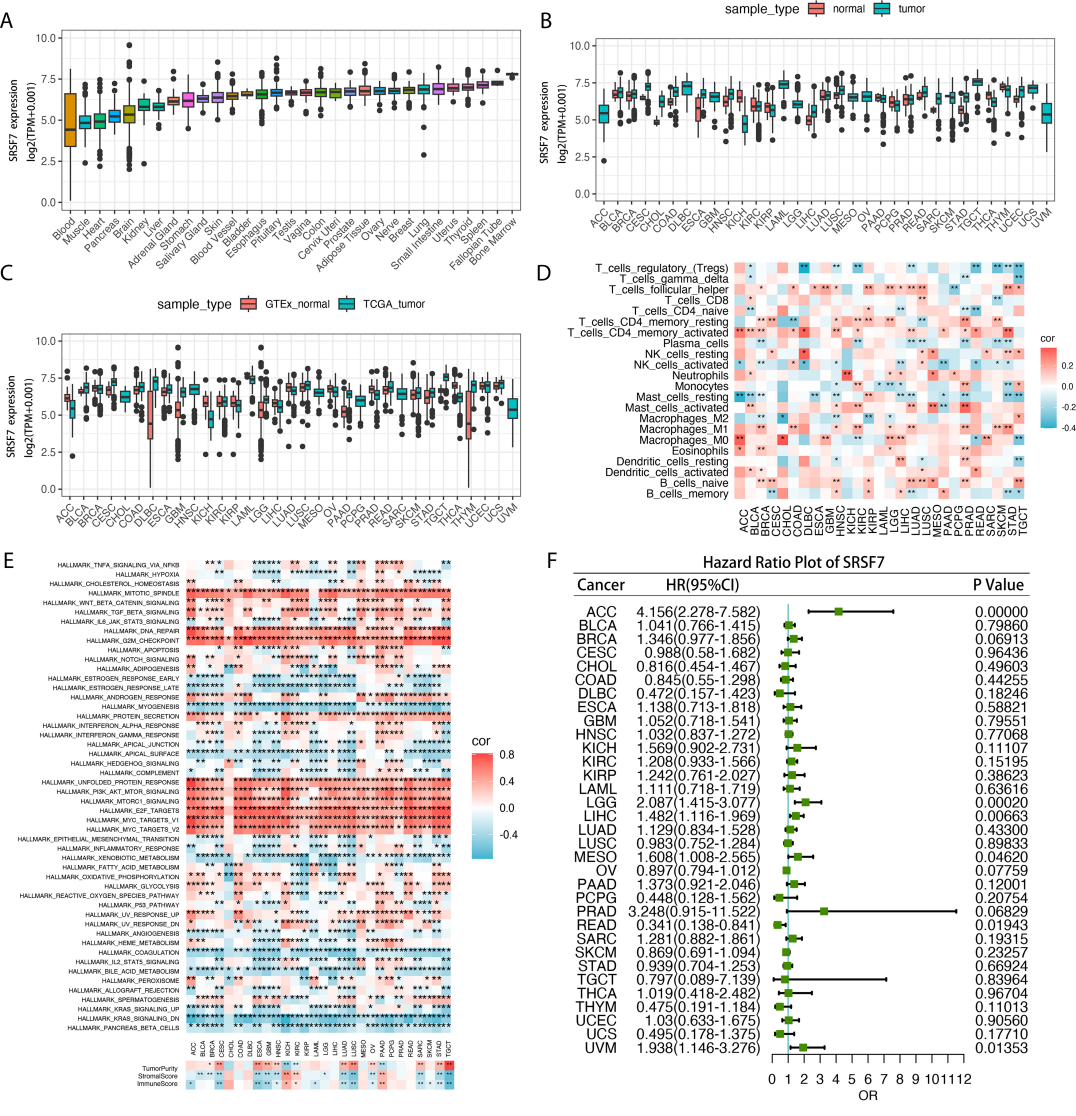
SRSF7 in pan-cancer analysis. **(A)** Pan-cancer SRSF7 mRNA expression in the TCGA dataset. The vertical axis represents the log2-transformed expression levels (log2(TPM+0.001)). **(B)** SRSF7 mRNA expression in normal and tumor samples from the TCGA dataset. The vertical axis represents the log2-transformed expression levels (log2(TPM+0.001)). **(C)** SRSF7 mRNA expression in normal samples from GTEx and tumor samples from the TCGA dataset. The vertical axis represents the log2-transformed expression levels (log2(TPM+0.001)). **(D)** Correlation analysis between pan-cancer SRSF7 expression and immune cell infiltration. **(E)** Correlation analysis between pan-cancer SRSF7 expression and Hallmark and immune infiltration pathways. **(F)** Forest plot showing the prognostic significance of SRSF7 across different tumors. *p < 0.05, **p < 0.01,***p < 0.001, ****p < 0.0001.

To further analyze the role of SRSF7 in other malignant tumors, we conducted a pan-cancer analysis of SRSF7. In this study, we first calculated the expression level of SRSF7 in cancer cells using the TCGA pan-cancer database (**Figure 7A**). The results showed that SRSF7 was universally overexpressed in various tissues and cancer cell lines, especially in bone marrow tissue. Subsequently, we evaluated the differential expression of SRSF7 between cancer and non-cancer tissue samples in the TCGA database (**Figure 7B**). Considering the limited number of normal samples in the TCGA database, we combined the GTEx and TCGA databases to conduct an in-depth study of SRSF7 expression in 27 types of tumors, revealing differential expression of SRSF7 in numerous cancers (**Figure 7C**). Furthermore, to explore the association between SRSF7 and the clinical prognosis of 33 types of cancer patients, we performed univariate analysis using the TCGA dataset. The forest plot revealed that SRSF7 significantly influenced the overall survival (OS) of several specific tumor types among 28 cancer types (**Figure 7F**), including UVM, READ, MESO, LGG, LIHC, and ACC. Through Hallmark gene set enrichment analysis, we found that SRSF7 significantly affected pathways such as DNA_REPAIR, G2M_CHECKPOINT, MITOTIC_SPINDLE, E2F_TARGETS, and MYC_TARGETS in the aforementioned cancers (**Figure 7E**). The regulation of these pathways is essential for the occurrence, development, and cell cycle regulation of cancer. Immune cell infiltration is crucial for the occurrence, development, and immune escape mechanisms of tumors. SRSF7 expression was highly correlated with pathways such as T_Cells_CD4_Memory_resting, T_Cells_CD4_Memory_activated, and T_cells_follicular_helper (**Figure 7D**). Additionally, we used the R package “Estimate” to evaluate the stromal score of each tumor sample. The top three cancers with the most significant positive correlation between SRSF7 expression levels and stromal score, immune score were KICH, KIRC, and PAAG. The positive correlation with tumor purity was observed in CESC, ESCA, TGCT, among others.

### 3.9 *In vitro* functional verification of SRSF7

**Figure 8:**
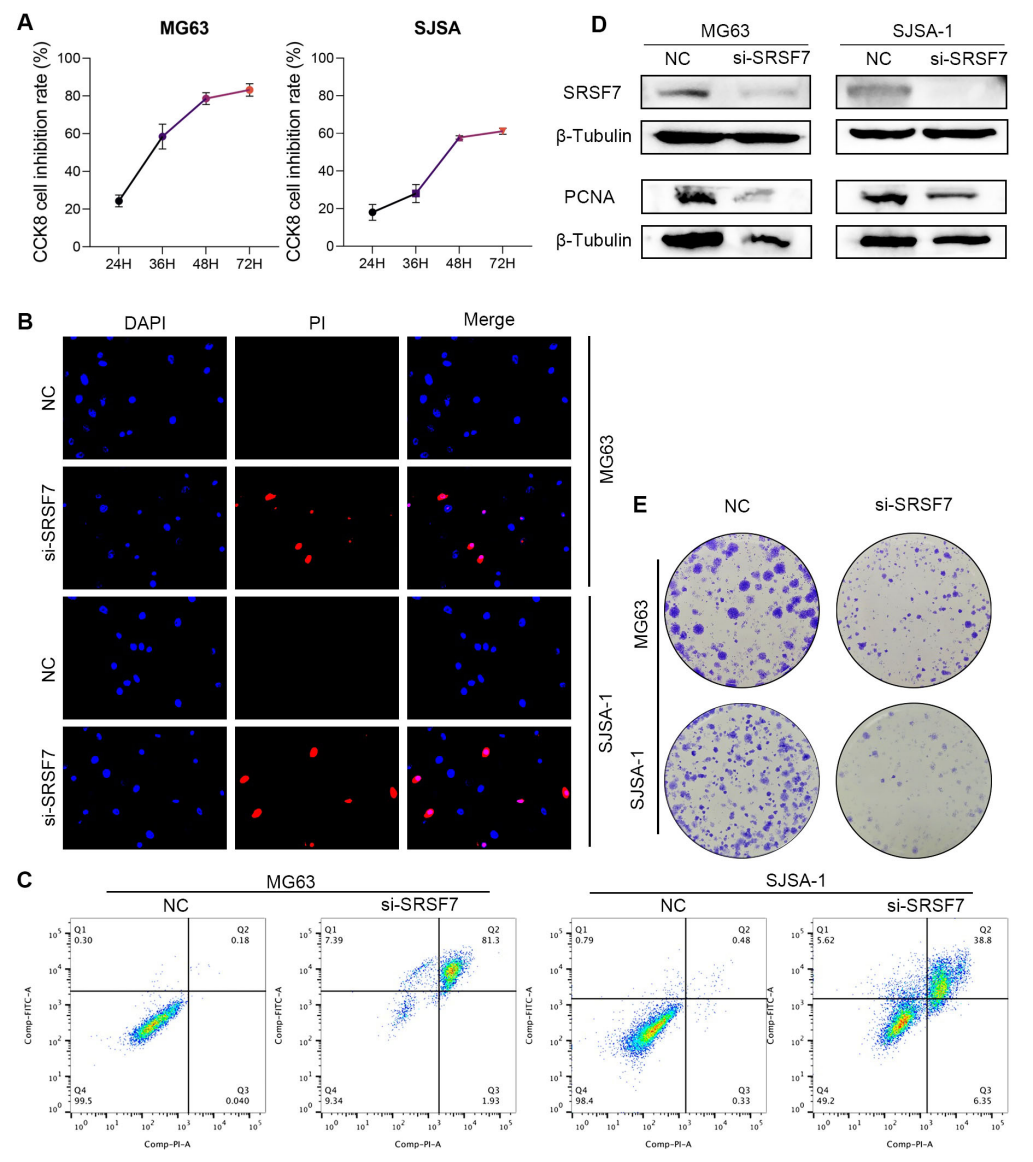
Regulation of osteosarcoma cell proliferation and apoptosis by SRSF7 **(A)** Absorbance at 450 nm wavelength after CCK8 treatment in different SRSF7 treatment groups at different time nodes. The more absorbance increased, the more cell proliferation. **(B)** DAPI & PI staining was performed on different SRSF7 treatment groups. Top to bottom were all cells in the field of view, apoptosing cells, and the composite of the above two images. The more pink cells, the more apoptosing cells. **(C)** Apoptosis levels in different SRSF7 treatment groups. **(D)** Protein expression levels of SRSF7 and PCNA in MG63 and SJSA-1 cells in SRSF7 knockdown groups (si-SRSF7) and Control group (NC). **(E)** Plate cloning was performed on different SRSF7 treatment groups.

In our previous study, we found that SRSF7 plays a significant regulatory role in osteosarcoma. Therefore, SRSF7 is expected to be a promising new therapeutic target in osteosarcoma treatment. si-SRSF7 was transfected into MG63 and SJSA-1 cell lines to investigate the effect of SRSF7 on the growth of osteosarcoma cell lines. The siRNA downregulation efficiency for SRSF7 was confirmed by Western blotting, with a knockdown efficiency of approximately 80% (**Figure 8D**). PCNA (Proliferating Cell Nuclear Antigen) was reported as a marker of cell proliferation, reflecting the proliferative capacity of the cells. β- Tubulin was used as the normalization reference gene for all Western blot analyses to ensure accurate quantification of protein levels. Each experiment was conducted in triplicate to ensure reproducibility and statistical significance. According to the CCK-8 assay results, downregulation of SRSF7 inhibited the viability of MG63 and SJSA-1 cell lines (**Figure 8A**). The colony formation assay results showed that downregulation of SRSF7 expression inhibited the colony-forming ability of MG63 and SJSA-1 cell lines (**Figure 8E**). Additionally, we performed flow cytometry apoptosis assays and PI staining to assess cell apoptosis and necrosis. We found that si-SRSF7 promoted apoptosis in these cells, with the majority of the cell population in the late apoptosis stage (Annexin V+ PI+). This suggests that SRSF7 knockdown leads to significant cell death, likely through both apoptotic and necrotic pathways (**Figures 8B, C**).

## 4 Discussion

Osteosarcoma is the most common malignant bone tumor. Serine/arginine-rich splicing factors (SRs) are an important class of splicing regulatory factors, typically comprising 12 members (SRSF1-12). They play crucial roles in post-transcriptional regulation of mRNA, affecting biological processes such as alternative splicing patterns, mRNA stability, and output. Aberrant expression of SRs can lead to changes in gene splicing patterns within tumor cells, thereby impacting various critical biological processes, including cell proliferation, metastasis, invasion, angiogenesis, and immune evasion (37–39). Therefore, by investigating the role of SRs in osteosarcoma development, we can better understand the importance of splicing regulation in osteosarcoma biology. In this study, we constructed a prognostic model based on serine/arginine-rich splicing factors (SRs) and predicted the survival of osteosarcoma patients. Through analysis of single-cell RNA sequencing data and application of AUCell enrichment analysis, we revealed the oncogenic pathways of SRs in osteosarcoma immune cells. Additionally, we described the regulatory role of SRSF7 in human pan-cancer.

In our study on the construction of a prognostic model for osteosarcoma, we found a correlation between six serine/arginine-rich splicing factors (SRs) and patient prognosis. Patients classified into the high-risk SRs group showed a significant decrease in survival rate, highlighting the crucial role of these SRs in the progression of osteosarcoma. Early research results also confirmed the role of SRs family members in tumor development. Specifically, studies have shown that SRSF1 influences the migration, invasion, proliferation, and apoptosis of osteosarcoma cells, while SRSF3 regulates ILF3 RNA splicing to control osteosarcoma growth (40, 41). Additionally, a significant increase in SRSF1 levels was observed in cervical cancer cells (42), revealing a correlation between the increased cytoplasmic levels of SRSF1 and early tumor progression (43). Overexpression of SRSF2 promoted proliferation in colorectal cancer cells, while inhibition of its expression prevented tumor formation (44). Similarly, the synergistic effect of SRSF4 with platinum-based drugs induced apoptosis in cancer cells (45). Overexpression of SRSF5 and SRSF7 in lung cancer and colorectal cancer tissues, and knockdown of SRSF7 induced apoptosis in colorectal and lung cancer cells (46, 47). Additionally, the interaction between c-Myc and SRSF10 has been shown to promote proliferation of breast cancer cells. Furthermore, in our enrichment analysis results, we found that inhibition of SRs was associated with the suppression of immune-related pathways such as Inflammatory response, Interferon GAMMA response, IL2/STAT5 signaling, and complement system. Previous studies have also suggested that SRs may promote cancer immune evasion by increasing the levels of immune checkpoint molecules (48). Moreover, through GO and KEGG enrichment analysis methods, we also observed the activation of RNA metabolism processes, organelle organization regulation, and transcriptional regulation pathways dependent on RNA polymerase II (33). The upregulation of these pathways revealed that SRs promote tumor cell proliferation, survival, and facilitate tumor evasion of host immune surveillance by regulating relevant signaling pathways.

It is increasingly recognized that enhancing host immunity may be an effective strategy for cancer treatment (49, 50). Regrettably, our understanding of the local immune characteristics within the tumor microenvironment remains quite limited. To gain a clearer understanding of the role of SRs in immune regulation, single-cell technologies have provided a method for characterizing the phenotype and function of tumor-infiltrating immune cells. We performed enrichment analysis of SRs activity using AUCell, successfully stratifying cells into Active and Inactive groups, facilitating a more comprehensive exploration of the regulatory functions of SRs. Differential analysis unveiled a strong concordance between the enrichment outcomes of these two groups and transcriptomic data. Specifically, we observed an upregulation of pathways involved in promoting cellular proliferation and growth, including E2F TARGETS, MYC TARGETS, and G2M CHECKPOINT, in the enrichment results (30, 51). Conversely, pathways related to angiogenesis, coagulation, epithelial-mesenchymal transition, TNF-α signaling, and response to hypoxic environments showed a downregulation trend. Research suggests that SRSF1 is commonly upregulated in cancer and serves as a direct target of Myc (52) .Additionally, enrichment analyses of pathways such as T_CELL_RECEPTOR_SIGNALING_PATHWAY, SPLICEOSOME, and CELL_CYCLE highlight their significant involvement in regulating critical biological processes including cellular immune response, splicing mechanisms, and cell cycle dynamics. Particularly noteworthy is the pronounced enrichment observed in T cells, underscoring the pivotal role of SRs in T cell regulation. Our analysis also identified significant differences in T cell behavior between the two groups stratified based on high and low-risk SRSF expression. Therefore, we can conclude that T cells exert significant regulatory effects within the context of SRs.

Therefore, with the aid of these significant findings, we have advanced our understanding of NK/T cell classification, categorizing them into two major subsets: CD8^+^ T cells and CD4^+^ T cells. Through enrichment analysis, we have further confirmed the involvement of SRSFs in suppressing immune response pathways. In order to elucidate the critical transcription factors underlying immune regulation, we identified transcription factors such as STAT2, TBX15, ETS1, and FOSL1, which exhibit significant regulatory roles. In particular, the STAT family, identified as potential therapeutic targets or immune checkpoint inhibitors, has shown importance in the treatment of various cancers (53, 54). Research by Govender highlights that STAT2 regulates IL-10 expression in CD4^+^ T cells in response to type I interferon (55). Additionally, TNF-α induced activation of NF-κB enhances the expression of TBX15 mRNA in cancer cells (56). Moreover, TBX15 also contributes to immune escape and metastasis by upregulating PD-L1 and enhancing the interaction between PTBP1 and FOSL1 (57).

To investigate immune regulatory changes in CD4^+^ and CD8^+^ T cells, we utilized the reference projection automated method of ProjecTILs. Through detailed annotation analysis of CD8^+^ T cells, we identified that those in an active state are primarily classified as GZMK^+^ Tex, terminal TEX, ISG^+^CD8^+^ T cells, and other subtypes. Similarly, in the analysis of CD4^+^ T cells, we observed an increase in the number of TNFRSF9^+^ Tregs CD4^+^ T cells in the Active state. These findings are consistent with previous research. Specifically, terminal exhausted T cells (TEX) are known to be enriched with tumor antigens, making them a key factor in tumor immune evasion (58). ISG+ dendritic cells (DCs) can activate CD8+ T cells and promote protective anti-tumor immune responses, even in the absence of conventional dendritic cell 1 (cDC1) (59). Additionally, the presence of Tn antigen in the tumor microenvironment (TME) has been found to suppress Th1 cell responses and induce T cells to produce interleukin-17 (IL-17), potentially contributing to immune evasion by tumor cells (60). The increased ratio of regulatory T cells (Tregs) to cytotoxic CD8^+^ T cells has been widely recognized as associated with poor prognosis. The immunosuppressive function of Tregs may hinder the attack of CD8^+^ T cells against tumor cells, thereby promoting tumor growth and dissemination (61). Further research indicates that an increase in the immunosuppressive subset of CD4^+^ T cells known as TNFRSF9^+^ Tregs contributes to immune evasion and T cell dysfunction in late-stage renal cell carcinoma (KIRC) (62).

We conducted in-depth analysis of the temporal changes of six serine/arginine-rich splicing factors (SRs) across different cell populations using the Monocle3 algorithm. Notably, these splicing factors exhibited higher expression levels during the NK/T cell stages, particularly concentrated in the early differentiation stages. Specifically, the increased expression of SRSF7, SRSF5, and SRSF10 in T cells was particularly notable, indicating their potential roles in T cell development and function.

To further validate the function of SRSF7, we conducted a series of *in vitro* cell experiments. Silencing SRSF7 gene expression significantly inhibited cell proliferation in osteosarcoma cell lines, as demonstrated by plate colony formation and CCK-8 assays. Flow cytometry analysis confirmed that this inhibition was due to the induction of apoptosis. Additionally, Western Blot analysis revealed changes in the expression of the proliferation marker PCNA, further supporting the regulatory role of SRSF7 in tumor cell proliferation.

Additionally, SRSF7 is not only involved in the progression of osteosarcoma but is also closely associated with UVM, READ, MESO, LGG, LIHC, ACC, immune cell infiltration, and immune pathways across multiple cancers. Our observations indicate that SRSF7 knockdown leads to a rapid progression to late apoptosis, as evidenced by Annexin V+ PI+ staining. Our study found that the enriched pathways in both the high-risk and low-risk SR groups are associated with TNF-α, hypoxia, and MYC, which are related to oxidative stress and inflammation (63). TNF-α is a pro-inflammatory cytokine that, upon binding to its receptors (TNFR1 and TNFR2), recruits FADD and Caspase-8 to form the Death-Inducing Signaling Complex (DISC), leading to Caspase-8 activation and apoptosis (64). Additionally, certain conditions, such as the presence of quercetin, can activate NFκB and COX2, resulting in necrotic cell death in the BT-474 cell line (65). Furthermore, after oxygen-glucose deprivation, the NFκB signaling pathway induces COX2, promoting cell death in wild-type astrocytes (66). The sustained activation of these inflammatory pathways can lead to excessive inflammation and significant stress or damage, causing cells to primarily die from necrosis and late apoptosis (67). These findings suggest that the rapid progression to late apoptosis observed in our study is closely related to the activation of inflammatory and oxidative stress pathways following SRSF7 knockdown.

Although our study validated the effectiveness of SRs in predicting the prognosis of osteosarcoma patients through *in vitro* cell experiments and highlighted the oncogenic role of SRSF7 in osteosarcoma cells, there are still some limitations that point to directions for future research. Firstly, although the role of SRSF7 has been validated at the *in vitro* cellular level, its effects in human tissue samples have not yet been confirmed. Additionally, *in vivo* tumorigenesis experiments in mouse models have not been conducted to further explore its mechanisms of action in living organisms. Furthermore, while we observed changes in apoptosis, the specific mechanisms underlying this process were not further validated through additional experiments. Lastly, besides SRSF7, other prognostically relevant genetic features should also be thoroughly validated at the cellular and molecular levels.

In this study, we constructed a model using Lasso regression to classify osteosarcoma patients into high and low-risk groups based on SRs gene expression, revealing significant differences in prognosis and tumor microenvironment. Single-cell RNA sequencing and AUCell enrichment analysis further showed that SRs are active in regulating T cell functions and immune evasion. Experimental results suggest that SRSF7 is a key factor influencing osteosarcoma proliferation and apoptosis. These findings provide a new perspective for predicting patient prognosis and highlight the potential of SRSFs, particularly SRSF7, as therapeutic targets in osteosarcoma.

## Data Availability

The datasets presented in this study can be found in online repositories. The names of the repository/repositories and accession number(s) can be found in the article/**Supplementary Material**.
